# Adult Medication-Free Schizophrenic Patients Exhibit Long-Chain Omega-3 Fatty Acid Deficiency: Implications for Cardiovascular Disease Risk

**DOI:** 10.1155/2013/796462

**Published:** 2013-02-27

**Authors:** Robert K. McNamara, Ronald Jandacek, Therese Rider, Patrick Tso, Yogesh Dwivedi, Ghanshyam N. Pandey

**Affiliations:** ^1^Department of Psychiatry, University of Cincinnati College of Medicine, Cincinnati, OH 45219-0516, USA; ^2^Department of Pathology, University of Cincinnati, Cincinnati, OH 45237, USA; ^3^Psychiatric Institute, Department of Psychiatry, University of Illinois at Chicago, Chicago, IL 60612, USA

## Abstract

Deficiency in long-chain omega-3 (LC*n* − 3) fatty acids, eicosapentaenoic acid (EPA, 20:5*n* − 3) and docosahexaenoic acid (DHA, 22:6*n* − 3), has been implicated in the pathoetiology of cardiovascular disease, a primary cause of excess premature mortality in patients with schizophrenia (SZ). In the present study, we determined erythrocyte EPA + DHA levels in adult medication-free patients SZ (*n* = 20) and age-matched healthy controls (*n* = 24). Erythrocyte EPA + DHA composition exhibited by SZ patients (3.5%) was significantly lower than healthy controls (4.5%, −22%, *P* = 0.007). The majority of SZ patients (72%) exhibited EPA+DHA levels ≤4.0% compared with 37% of controls (Chi-square, *P* = 0.001). In contrast, the omega-6 fatty acid arachidonic acid (AA, 20:4*n* − 6) (+9%, *P* = 0.02) and the AA:EPA + DHA ratio (+28%, *P* = 0.0004) were significantly greater in SZ patients. Linoleic acid (18:2*n* − 6) was significantly lower (−12%, *P* = 0.009) and the erythrocyte 20:3/18:2 ratio (an index of delta6-desaturase activity) was significantly elevated in SZ patients. Compared with same-gender controls, EPA + DHA composition was significantly lower in male (−19%, *P* = 0.04) but not female (−13%, *P* = 0.33) SZ patients, whereas the 20:3/18:2 ratio was significantly elevated in both male (+22%, *P* = 0.008) and female (+22%, *P* = 0.04) SZ patients. These results suggest that the majority of SZ patients exhibit low LC*n* − 3 fatty acid levels which may place them at increased risk for cardiovascular morbidity and mortality.

## 1. Introduction

Patients with schizophrenia (SZ) have two- to three-fold higher mortality rates compared with the general population, corresponding to an average 15-year reduction in life expectancy, and cross-sectional epidemiological studies have found that cardiovascular disease is a primary cause of excess premature mortality in SZ patients [1–6]. The etiology of elevated cardiovascular risk in SZ is likely multifactorial, potentially involving excessive smoking and alcohol use, lack of exercise, and poor diets [7, 8]. Moreover, second generation antipsychotic (SGA) medications are associated with cardiovascular risk factors including dyslipidemia, metabolic syndrome, and weight gain [9–13], though these risk factors have also been reported in SGA-naïve first-episode SZ patients [14–16]. Together, these data highlight an urgent need to identify risk and resilience factors associated with elevated cardiovascular disease risk in SZ.

An emerging body of evidence suggests that low levels of long-chain omega-3 (LC*n *− 3) fatty acids, principally eicosapentaenoic acid (EPA, 20:5*n *− 3) and docosahexaenoic acid (DHA, 22:6*n *− 3), are a modifiable risk factor for cardiovascular disease [17]. Mammals are entirely dependent on their diet to obtain LC*n *− 3 fatty acids, and erythrocyte EPA + DHA composition is positively correlated with habitual dietary LC*n *− 3 fatty acid intake [18–20]. It is relevant, therefore, that in Japan, where the prevalence of cardiovascular mortality among men is 6-fold lower than in the United States (USA) [21], annual seafood consumption is 3-fold greater than in the USA [22] and is associated with ~2-fold greater erythrocyte EPA + DHA levels [23, 24]. Prospective studies have found that erythrocyte EPA + DHA composition (“omega-3 index”) ≤4% of total fatty acids is associated with a 10-fold greater risk for sudden cardiac arrest compared with >8% EPA + DHA composition [25]. Additionally, patients with acute coronary syndrome (ACS) exhibit significantly lower erythrocyte EPA + DHA levels compared with healthy controls [26]. Erythrocyte EPA + DHA composition is positively correlated with human myocardium biopsy EPA + DHA composition [27], and a cardiac biopsy study found that patients that died from cardiovascular disease exhibited lower heart DHA levels compared with patients dying from noncardiac causes [28]. Together, these and other data suggest that low dietary LC*n *− 3 fatty acid intake represents a modifiable risk factor for cardiovascular disease in the general population, and that erythrocyte EPA + DHA composition represents a valid risk biomarker for cardiovascular morbidity and mortality.

Several previous case-control studies have observed lower erythrocyte DHA levels in medication-naive SZ patients [29–34]. Emerging clinical evidence suggests that chronic exposure to SGA medications partially normalize erythrocyte DHA deficits in first-episode SZ patients [29, 30, 32, 34] and in the postmortem prefrontal cortex of SZ patients [35]. Preclinical evidence further suggests that chronic treatment with SGA medications increases rat erythrocyte and heart EPA + DHA composition by augmenting biosynthesis [36, 37]. Furthermore, preclinical and clinical evidence suggests that ovarian hormones augment LC*n *− 3 fatty acid biosynthesis and erythrocyte DHA composition [38–41], and that erythrocyte [31] and postmortem brain [35] DHA deficits are more robust in male than female SZ patients. The primary objective of the present study was to compare erythrocyte EPA + DHA composition in adult medication-free male and female SZ patients and healthy controls. Based on cross-sectional evidence that SZ patients are at higher risk than the general population for developing cardiovascular disease, our specific prediction was that erythrocyte EPA + DHA composition would be significantly lower in SZ patients.

## 2. Materials and Methods

### 2.1. Subject Demographics

These studies were conducted in hospitalized male and female patients with SZ (*n* = 20) admitted to the Psychiatric Clinical Research Center, as part of the General Clinical Research Center (GCRC), University of Illinois at Chicago. Healthy adult male and female controls with no history of psychiatric illness (*n* = 24) were recruited from the greater Chicago area. A comparison of group demographic variables is presented in Table 1. Patients were kept medication-free for up to 2 weeks prior to blood collection to permit sufficient washout of antipsychotic medications. Data regarding smoking status, diet, and body mass index were not obtained. This study was approved by the Institutional Review Board of the University of Illinois at Chicago.

### 2.2. Erythrocyte Fatty Acid Composition

Whole venous blood (40 mL) was collected into tubes containing 4 mL of sodium citrate (3.8%) and centrifuged at 210 ×g for 15 min at 4°C. Plasma and the platelet-rich interface were removed, and erythrocytes were washed twice with 0.9% saline and stored at −80°C. Erythrocyte membrane total fatty acid composition was determined with a Shimadzu GC-2014 (Shimadzu Scientific Instruments Inc., Columbia, MD, USA) using the saponification and methylation procedure described previously [42]. Analysis of fatty acid methyl esters was based on area under the curve calculated with Shimadzu Class VP 4.3 software. Fatty acid identification was based on retention times of authenticated fatty acid methyl ester standards (Matreya LLC Inc., Pleasant Gap, PA, USA). Data are expressed as weight percent of total fatty acids (mg fatty acid/100 mg fatty acids). All samples were processed by a technician blinded to group identity. Our primary measure of interest was EPA + DHA. We also determined erythrocyte indices of delta9-desaturase activity (stearoyl-CoA desaturase, 16:1/16:0 and 18:1/18:0 ratios), delta6-desaturase (20:3*n *− 6/18:2*n *− 6), delta5-desaturase (20:4*n *− 6/20:3*n *− 6), and an index of “*de novo *lipogenesis” (16:0/18:2).

### 2.3. Statistical Analysis

Group differences in erythrocyte fatty acid composition were evaluated with unpaired *t*-tests (2-tailed) and corrected for multiple comparisons (*α* = 0.01). The distribution of fatty acids was examined for normality using Bartlett's test. Categorical data were evaluated with the Chi-square test. Parametric (Pearson) correlation analyses were performed to determine relationships between fatty acids and fatty acid ratios (2-tail, *α* = 0.05). Effect size was calculated using Cohen's *d*. Analyses were performed using GB-STAT (V.10, Dynamic Microsystems, Inc., Silver Springs, MD, USA).

## 3. Results

Erythrocyte samples from two SZ patients were excluded from the final analyses because of evidence for gross abnormalities in membrane fatty acid composition, including the absence of DHA. The fatty acid composition of erythrocytes from SZ patients (*n* = 18) and healthy controls (*n* = 24) are presented in Table 2. Consistent with our *a priori *hypothesis, erythrocyte EPA + DHA composition (“omega-3 index”) was significantly lower in SZ patients compared with controls (−22%, *P* = 0.007, *d* = 0.89). This difference was attributable to lower DHA (−21%, *P* = 0.01) and EPA composition (−24%, *P* = 0.12). There were no significant group differences for saturated fatty acids (16:0, 18:0) or monounsaturated fatty acids (16:1*n *− 7, 18:1*n *− 7, 18:1*n *− 9). Among the omega-6 fatty acids, linoleic acid (18:2*n *− 6) was significantly lower in SZ patients (−12%, *P* = 0.009, *d* = 0.86), and arachidonic acid (AA, 20:4*n *− 6) (+9%, *P* = 0.01, *d* = 0.77) and docosapentaenoic acid (22:5*n *− 6) (+19%, *P* = 0.003, *d* = 0.99) were significantly greater in SZ patients. Erythrocyte AA/DHA (+27%, *P* = 0.0007, *d* = 1.1) and AA/EPA + DHA (+28%, *P* = 0.0004, *d* = 0.87) ratios, but not the AA/EPA ratio (*P* = 0.25), were significantly greater in SZ patients.

**Figure 1:**
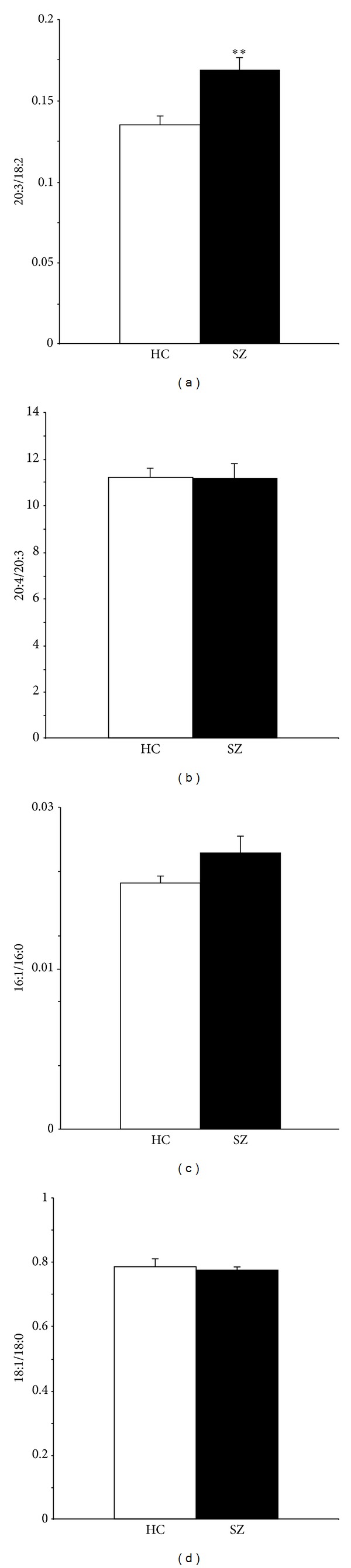
Comparison of the 20:63/18:2 ratio, an index of delta6-desaturase activity (a), the 20:4/20:3 ratio, an index of delta5-desaturase activity (b), and the 16:1/16:0 (c) and 18:1/18:0 (d) ratios, indices of delta9-desaturase activity, in healthy controls (HC, *n* = 24) and SZ patients (*n* = 18). Values are group mean ± SEM. **P ≤ 0.001 versus healthy controls.

The erythrocyte 20:3/18:2 ratio, an index of delta6-desaturase activity, was significantly greater in SZ patients (+20%, *P* = 0.001, *d* = 1.1) (Figure 1(a)), whereas the 20:4/20:3 ratio, an index of delta5-desaturase activity, did not differ between groups (*P* = 0.96) (Figure 1(b)). There were no significant group differences in the 16:1/16:0 (*P* = 0.29) (Figure 1(c)) and 18:1/18:0 (*P* = 0.83) ratios (Figure 1(d)), indices of delta9-desaturase (stearoyl-CoA desaturase) activity. The erythrocyte 16:0/18:2 ratio, an index of *do novo* lipogenesis, was significantly greater in SZ patients (+16%, *P* = 0.01). Among SZ patients (*n* = 18), EPA + DHA was not significantly correlated with the 20:3/18:2 ratio (*r* = −0.12, *P* = 0.64), the 20:4/20:3 ratio (*r* = +0.02, *P* = 0.95), the 16:1/16:0 ratio (*r* = −0.17, *P* = 0.64), the 18:1/18:0 ratio (*r* = +0.07, *P* = 0.78), or the 18:2/16:0 ratio (*r* = −0.38, *P* = 0.12). Among healthy controls (*n* = 24), EPA + DHA was not significantly correlated with the 20:3/18:2 ratio (*r* = −0.12, *P* = 0.56), the 20:4/20:3 ratio (*r* = +0.11, *P* = 0.59), the 16:1/16:0 ratio (*r* = −0.06, *P* = 0.83), the 18:1/18:0 ratio (*r* = +0.12, *P* = 0.58), or the 18:2/16:0 ratio (*r* = +0.14, *P* = 0.51). Among all subjects (*n* = 42), EPA + DHA was inversely correlated with the 20:3/18:2 ratio (*r* = −0.29, *P* = 0.05) and was not correlated with other ratios. Among SZ patients (*n* = 18), positive and negative symptom scale (PANSS) total scores were not significantly correlated with DHA (*P* = 0.43), EPA + DHA (*P* = 0.19), AA/EPA + DHA (*P* = 0.85), or the 20:3/18:2 ratio (*P* = 0.84).

**Figure 2:**
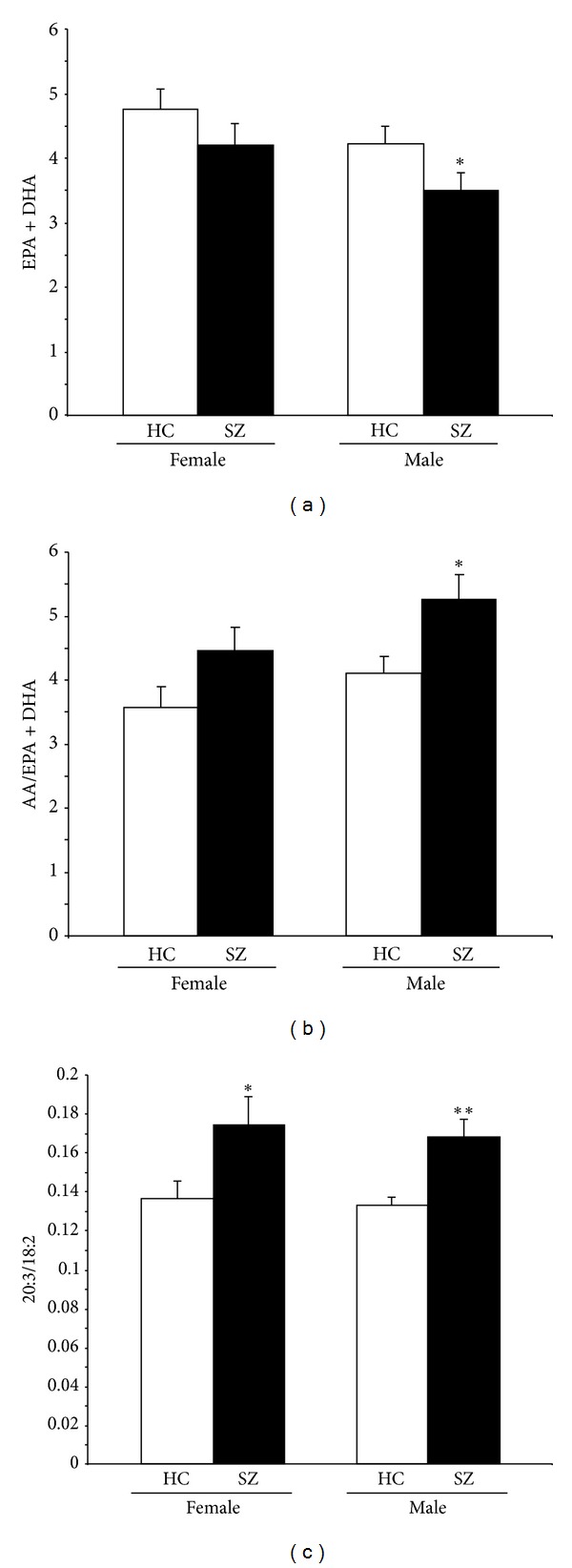
The erythrocyte Omega-3 Index (EPA + DHA) (a), the ratio of arachidonic acid (20:4*n *− 6) to the Omega-3 Index (EPA + DHA) (b), and the 20:3/18 ratio, an index of delta6-desaturase activity (c), in female and male and healthy controls (HC) and SZ patients. Values are group means ± SEM. *P ≤ 0.05, **P ≤ 0.01 versus same-gender healthy controls.

Compared with same-gender controls, EPA + DHA composition was significantly lower in male (−19%, *P* = 0.04, *d* = 0.9) but not female (−13%, *P* = 0.33) SZ patients (Figure 2(a)), and the AA/EPA + DHA ratio was significantly higher in male (+22%, *P* = 0.03, *d* = 1.0) but not female (+19%, *P* = 0.16) SZ patients (Figure 2(b)). Compared with same-gender controls, the 20:3/18:2 ratio was significantly greater in both male (+22%, *P* = 0.008, *d* = 1.3) and female (+22%, *P* = 0.04, *d* = 1.0) SZ patients (Figure 2(c)). In healthy controls, EPA + DHA composition (*P* = 0.35), the AA/EPA + DHA ratio (*P* = 0.12), and the 20:3/18:2 ratio (*P* = 0.78) did not differ between males and females.

**Figure 3:**
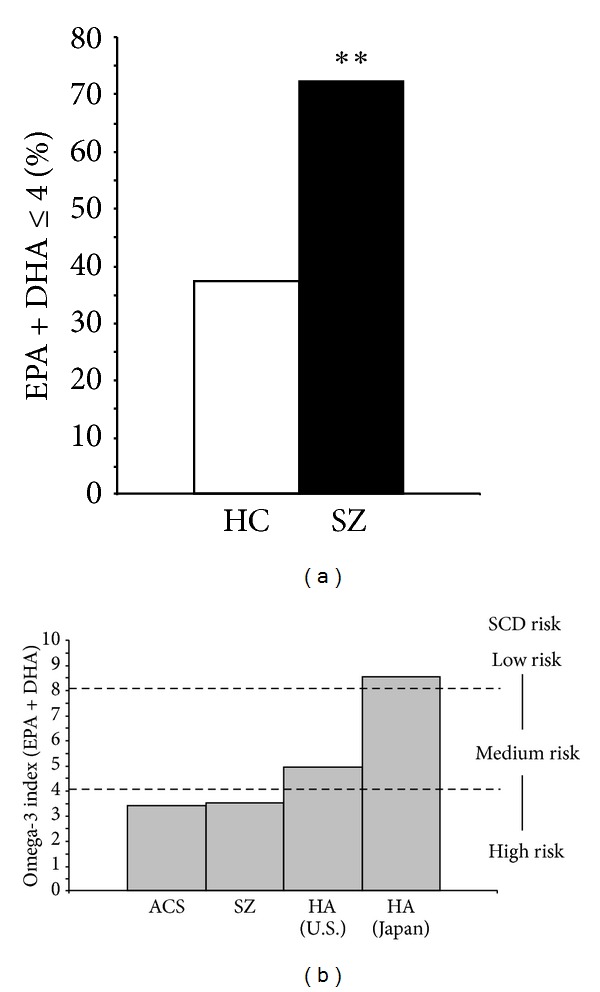
(a) The percentage of healthy controls (37%) and SZ patients (72%) with an omega-3 index (EPA + DHA) of ≤4.0%. (b) Comparison of the omega-3 index in adult SZ patients residing in the USA (present study), adult US patients with acute coronary syndrome (ACS, *n* = 768, from [26]), healthy adults (HA) residing in the USA (*n* = 163, from [23]), and healthy adults residing in Japan (*n* = 456; from [24]). Proposed risk zones for sudden cardiac death (SCD) derived from prior prospective longitudinal evidence [25] are indicated **P = 0.001 (Chi-Square).

The majority of SZ patients (72%) exhibited erythrocyte EPA + DHA levels ≤4.0% compared with 37% of controls (Chi-square, *P* = 0.001) (Figure 3(a)), indicating a 2-fold greater number of SZ patients exhibit an erythrocyte EPA + DHA level of ≤4.0% compared with controls. A comparison of the omega-3 index in adult SZ patients residing in the USA, adult US patients with acute coronary syndromes (ACS, *n* = 768, from [26]), healthy adults (HA) residing in the USA (*n* = 163, from [23]), and healthy adults residing in Japan (*n* = 456; from [24]) is presented in Figure 3(b).

## 4. Discussion

There is now substantial evidence that lower erythrocyte EPA + DHA composition (the “omega-3 index”) is a modifiable risk factor for cardiovascular disease [17], a primary cause of excess premature mortality in patients with SZ. Consistent with our *a priori *hypothesis, erythrocyte EPA + DHA composition exhibited by SZ patients (3.5% ± 1.1%) was significantly lower than healthy controls (−22%, 4.5% ± 1.1%, *P* = 0.007). These findings are consistent with other cross-sectional studies demonstrating that SZ is characterized by LC*n *− 3 fatty acid deficits [29–34]. Erythrocyte EPA + DHA composition exhibited by healthy controls is similar to that previously reported in a larger cohort of healthy subjects residing in the U.S. (4.9% ± 2.1%) [23]. Prospective studies suggest that erythrocyte EPA + DHA composition of ≤4% is associated with a 10-fold greater risk for sudden cardiac death compared with ≥8% [25]. In the present study, a greater number (2-fold) of SZ patients exhibit an erythrocyte EPA + DHA level of ≤4.0% compared with healthy controls. This biomarker therefore suggests that the majority of SZ patients in the present study are at elevated risk for cardiovascular morbidity and mortality. Lastly, erythrocyte EPA + DHA composition was significantly lower in male (−19%, *P* = 0.04) but not female (−13%, *P* = 0.33) SZ patients compared with same-gender controls. 

This study has three principal limitations. First, data regarding habitual dietary LC*n *− 3 fatty acid intake was not available to evaluate its contribution to the erythrocyte EPA + DHA deficits observed in SZ patients. However, several studies have found that erythrocyte EPA + DHA composition is highly positively correlated with habitual dietary EPA + DHA intake [18–20, 23, 24] and that supplementing the diet with LC*n *− 3 fatty acids increases erythrocyte EPA + DHA composition in SZ patients [43]. Second, data regarding cigarette smoking status was not available, and some prior studies [44–46], but not others [23, 47], have observed an inverse relationship between cigarette smoking and erythrocyte EPA and DHA composition in different populations. However, a radio-tracer study found that cigarette smoking was associated with greater EPA + DHA biosynthesis and plasma availability [48], and plasma EPA + DHA is positively correlated with erythrocyte EPA + DHA composition [25]. While the objective of this study was not to determine the cause of the EPA + DHA deficits, it remains possible that the erythrocyte EPA + DHA deficits observed in SZ patients are associated with dietary EPA + DHA insufficiency and/or cigarette smoking. Third, the small number of SZ patients used in the present study may not be representative of all patients with SZ. However, the results of the present study are very consistent with prior case-control studies [29–34], and effect sizes were typically large (*d* = 0.89 − 1.1). Nevertheless, additional studies are warranted to replicate and extend these findings in a different sample of SZ patients.

Prior preclinical and clinical evidence suggests that ovarian hormones augment LC*n *− 3 fatty acid biosynthesis and erythrocyte EPA + DHA composition [38–41], and reduced levels of circulating estrogen levels were observed in male first-episode psychotic patients [49]. Consistent with prior studies [23, 31], erythrocyte EPA + DHA composition did not differ between healthy male and female controls. However, we did find compared with same-gender controls that EPA + DHA composition was significantly lower in male (−19%, *P* = 0.04) but not female (−13%, *P* = 0.33) SZ patients. This finding is consistent with a prior study reporting that male (−21%) but not female (−12%) medication-naïve first-episode psychotic patients exhibited significant erythrocyte EPA + DHA deficits compared with same-gender controls [31]. This is also consistent with a prior study finding that male (−27%) but not female (−2%) SZ patients exhibit significant DHA deficits in postmortem prefrontal cortex [35]. It is relevant therefore that SGA-treated male SZ patients exhibit a 1.5-fold greater 10-year cardiovascular risk compared with female SZ patients [9], though other epidemiological data suggest that cardiovascular disease is the primary cause of excess mortality in female SZ patients whereas suicide is the primary cause of excess mortality in male SZ patients [5]. In view of prior evidence that low DHA levels are also associated with increased suicide risk [50, 51], elucidation of the interrelationship between circulating estrogen levels, tissue DHA concentrations, and cardiovascular and suicide risk may provide important insight into mechanisms contributing to higher premature mortality rates in male and female SZ patients.

Prior case-control studies have observed lower erythrocyte 18:2*n *− 6 levels in patients with acute coronary syndromes [52], and the present study found lower erythrocyte 18:2*n *− 6 levels in SZ patients. While this deficit may be due to lower 18:2*n *− 6 intake [8], it may also be a consequence of elevated liver delta6-desturase activity for which 18:2*n *− 6 is a substrate. For example, we previously reported that elevated liver delta6-desturase activity and expression in response to dietary *n *− 3 fatty acid deficiency was associated with lower erythrocyte and liver 18:2*n *− 6 levels despite comparable dietary 18:2*n *− 6 intake [53]. In the present study, the 20:3/18:2 ratio was significantly elevated in both male and female SZ patients, though the 20:3/18:2 ratio was not correlated with EPA + DHA in SZ patients. Taken in conjunction with our previous finding that delta6-desturase (FADS2) mRNA expression is significantly elevated in the frontal cortex of male and female SZ patients [54], these data suggest that male and female SZ patients both exhibit elevated delta6-desturase activity. Importantly, elevated indices of delta6-desturase activity were previously found to be positively associated with dyslipidemia, insulin resistance and obesity [55, 56], elevated proinflammatory signaling [57–59], and cardiovascular disease morbidity and mortality [60–62]. These data suggest that elevated delta-6 desaturase activity may also contribute risk for cardiovascular disease in male and female SZ patients.

The low EPA + DHA status of SZ patients may also have implications for resilience to SGA-induced hypertriglyceridemia. For example, concomitant treatment with EPA [63] or EPA + DHA [64] significantly decreased elevated triglyceride levels in SZ patients treated with the SGA clozapine. We recently reported that dietary-induced LC*n *− 3 fatty acid insufficiency leading to deficits in erythrocyte and liver EPA + DHA composition significantly augmented SGA-induced triglyceride accumulation (hepatic steatosis) and plasma triglyceride levels in rats [65]. Human erythrocyte and liver EPA + DHA levels are positively correlated [66], and patients with hepatic steatosis and/or obesity exhibit significantly lower liver and erythrocyte EPA + DHA levels [66–68]. Interestingly, unlike SZ patients residing in the USA, SGA-treated SZ patients residing in Japan, where erythrocyte EPA + DHA levels are ~2-fold greater than the USA [23, 24], do not exhibit hypertriglyceridemia or obesity [69]. Moreover, cardiovascular disease is a leading cause of excess mortality for SZ patients residing in western countries [1–6] but not Japan [70]. Together, these data suggest that the low LC*n *− 3 fatty acid status observed in SZ patients residing in western countries may represent a modifiable risk factor for SGA-induced weight gain, hypertriglyceridemia, and associated risk for developing cardiovascular disease. 

The present findings also have implications for brain function and associated psychopathology [71]. DHA is the primary LC*n *− 3 fatty acid in mammalian brain, and nonhuman primate [72] and human postmortem [73] studies suggest that cortical and erythrocyte DHA levels are positively correlated. We reported that SZ patients exhibit significant postmortem prefrontal cortex DHA deficits [35], and emerging evidence from magnetic resonance imaging studies suggest that peripheral (erythrocyte, plasma phospholipids) DHA status is correlated with resting and functional cortical activity [74, 75] and indices of membrane turnover [76]. Moreover, prior studies have found that erythrocyte DHA levels are inversely correlated with negative symptom severity in SZ patients [29, 34, 43], and correction of DHA deficiency reduced positive and negative symptom severity and delayed or prevented the onset of psychosis in ultra high-risk adolescents [77]. In the present study, erythrocyte DHA was not significantly correlated with PANSS total score, though this may be due to limited power. It is also relevant that a controlled trial found that adjunctive treatment with EPA accelerated treatment response and permitted a 20% reduction in SGA dose in first-episode psychotic patients [78].

In summary, the present case-control study demonstrates that medication-free adult SZ patients exhibit significant erythrocyte EPA + DHA deficits. Based on prior cross-sectional and prospective evidence, the present data suggest that the low EPA + DHA status exhibited by SZ patients, particularly male SZ patients, places them at increased risk for developing acute coronary syndromes and sudden cardiac arrest, as well as SGA-induced hypertriglyceridemia and hepatic steatosis. It is also noteworthy that EPA + DHA deficits are not unique to SZ, and other psychiatric disorders associated with excess premature mortality due in part to cardiovascular disease, including major depressive disorder and bipolar disorder [79], are also associated with significant erythrocyte EPA + DHA deficits [42]. Because the erythrocyte EPA + DHA deficits exhibited by SZ patients can be corrected by dietary LC*n *− 3 fatty acid supplementation, this represents a modifiable risk factor. Indeed, the present data add to a growing body of evidence that supports treating SZ patients with LC*n *− 3 fatty acids in an effort to reduce cardiovascular morbidity and mortality, as well as improve SGA tolerability and overall illness course. Increasing erythrocyte EPA + DHA composition to ≥8%, which is associated with the greatest protection from cardiovascular morbidity and mortality, would represent an appropriate target and is achievable with a daily EPA + DHA dose of ~2.0 g/d [19].

## Figures and Tables

**Table 1:** Subject demographics.

Variable	HC	SZ	*P* value^1^
Age (years), mean ± SD	32.5 ± 9.7	29.2 ± 8.2	0.25
Gender	10 M, 14 F	12 M, 6 F	
Race	5 AS, 6 AA, 1 H, 12 C	12 AA, 1 H, 5 C	
PANSS (Total)	—	62.1 ± 19.4	

^1^ *t*-tests (2-tail).

AS: Asian, AA: African American, H: Hispanic, C: Caucasian.

PANSS: positive and negative symptom scale.

**Table 2:** Erythrocyte fatty acid composition.

Fatty acid^1^	HC	SZ	*P* value^2^
Saturated fatty acids			
Palmitic acid (16:0)	16.9 ± 0.21	17.8 ± 0.57	0.166
Stearic acid (18:0)	16.1 ± 0.4	16.3 ± 0.51	0.735
Monounsaturated fatty acids			
Palmitoleic acid (16:1*n* − 7)	0.33 ± 0.01	0.37 ± 0.04	0.235
Vaccenic acid (18:1*n* − 7)	1.25 ± 0.03	1.36 ± 0.04	0.024
Oleic acid (18:1*n* − 9)	12.3 ± 0.24	12.5 ± 0.29	0.509
Polyunsaturated fatty acids			
Linoleic acid (18:2*n* − 6)	11.23 ± 0.33	9.94 ± 0.33	0.009
*γ*-Linoleic acid (18:3*n* − 6)	nd	nd	
Homo-*γ*-linolenic (20:3*n* − 6)	1.49 ± 0.06	1.69 ± 0.07	0.034
Arachidonic acid (AA, 20:4*n* − 6)	16.1 ± 0.45	17.6 ± 0.31	0.014
Docosatetraenoic acid (22:4*n* − 6)	3.9 ± 0.17	4.1 ± 0.19	0.276
Docosapentaenoic acid (22:5*n* − 6)	0.74 ± 0.03	0.91 ± 0.04	0.003
*α*-Linolenic acid (18:3*n* − 3)	nd	nd	
Eicosapentaenoic acid (EPA, 20:5*n* − 3)	0.42 ± 0.06	0.28 ± 0.04	0.091
Docosapentaenoic acid (22:5*n* − 3)	2.11 ± 0.09	2.11 ± 0.06	0.944
Docosahexaenoic acid (DHA, 22:6*n* − 3)	4.12 ± 0.21	3.26 ± 0.25	0.012
EPA + DHA (omega-3 index)	4.54 ± 0.23	3.54 ± 0.27	0.007
Ratios			
AA:EPA	47.1 ± 4.64	55.7 ± 5.21	0.2361
AA:DHA	4.14 ± 0.23	5.66 ± 0.36	0.0007
AA:EPA + DHA	3.75 ± 0.21	5.22 ± 0.35	0.0004
LC*n* − 6/LC*n* − 3	3.46 ± 0.15	4.35 ± 0.19	0.0007

^1^Weight percent total fatty acids (g/100 g) expressed as mean ± SEM.

^2^ *P* values from the *t*-tests (2-tail).

nd: not detected (below the limit of reliable detection).
